# An experimental study on the pollen particle blocking efficacy of a barrier nasal mask

**DOI:** 10.3389/fmed.2025.1681440

**Published:** 2025-10-16

**Authors:** Yan Zhang, Na Lu, Yuxiang Zhao, Jinping Wang

**Affiliations:** ^1^Operating Room, Shaanxi Provincial People's Hospital, Xi'an, China; ^2^Department of Otolaryngology, Shaanxi Provincial People's Hospital, Xi'an, China

**Keywords:** barrier nasal mask, bionic nasal hair, allergen-blocking gel, 3D-printed nasal cavity model, allergic rhinitis

## Abstract

**Objective:**

To explore the blocking effect of a barrier nasal mask composed of bionic nasal hair combined with a blocking gel on allergen particles in a 1:1 3D-printed nasal cavity model and to provide new ideas for the clinical prevention and treatment of allergic rhinitis.

**Methods:**

A 1:1 scale 3D-printed nasal cavity model was constructed, and dust-free paper was placed at specific anatomical locations within the model. The experimental group was defined as those wearing a nasal mask, whereas the control group did not wear a nasal mask. A simple breathing bag was used to simulate normal respiration, and a pneumatic nebulizer was employed to introduce stained *Artemisia annua* pollen. The simulated breathing experiments were conducted for 15 min and 30 min. The degree of staining on the dust-free paper in both groups was observed and scored.

**Results:**

At 15 min, the median (25th, 75th percentiles) total scores for all anatomical sites in the nonblocking group and blocking group were 3 (2, 4) and 0 (0, 1), respectively (*Z* = −9.094, *p* < 0.001). At 30 min, the total scores of the two groups were 4 (2, 5) and 1 (0, 2), respectively (*Z* = −9.062, *p* < 0.001). Additionally, the comparison of scores at all other individual anatomical sites revealed *p* < 0.001.

**Conclusion:**

This barrier nasal mask can effectively reduce pollen particle deposition at various anatomical sites in the nasal cavity. The crossover test using the same model verified the reliability of its blocking efficacy, which suggests that it is a potential innovative intervention for the prevention of allergic rhinitis.

## 1 Introduction

Epidemiological surveys have shown that the incidence of allergic rhinitis (AR) has increased over the past few decades. In Europe, the average prevalence of AR is 20.9% (1). In China, the prevalence of AR has risen significantly, from 11.1% in 2005 to 17.6% in 2022, with notable regional variations. Specifically, owing to its unique and extensive plant cultivation, northwest China has the highest confirmed prevalence of pollen-induced AR, reaching 31.4% (2). Pollen-induced rhinitis is a major chronic inflammatory respiratory disease in this region that exerts severe impacts on patients’ quality of life and socioeconomic development.

Allergic rhinitis is an IgE-mediated type I allergic reaction with a complex pathogenesis involving immune responses triggered by exposure to allergens in individuals with atopic diathesis (3). The core of AR lies in the interaction between atopic constitution and allergen exposure, a key factor whose role in disease initiation and progression has been confirmed by numerous studies (4). According to the WHO guidelines for AR management, environmental control and allergen avoidance—two components of the “four-in-one” principle—aim to reduce allergen entry into the nasal cavity, thereby lowering the risk of AR exacerbation (5, 6). However, the practical implementation of environmental control and allergen avoidance faces multiple challenges with extremely low feasibility (7). Relevant studies have demonstrated that although a series of environmental control measures (e.g., frequent pet cleaning, the use of impermeable bedding covers, and air filtration) can effectively reduce indoor allergen levels, they fail to significantly alleviate symptoms or improve the quality of life of AR patients. Currently, wearing masks is a common method to block allergens (8). Research has indicated that during the COVID-19 pandemic, pollen-allergic patients experienced significant relief of nasal and ocular allergic symptoms due to mandatory mask wearing (9). However, masks with effective allergen-blocking properties, such as N95 masks, have obvious drawbacks in practical use. These masks severely impede respiration and may even reduce blood oxygen saturation (10). For patients already experiencing a strong sense of suffocation due to AR-related nasal congestion, wearing these masks exacerbates their discomfort. Consequently, the usage rate of these effective allergen-blocking masks in daily life is low (11). Although some nasal congestion-relief products are designed to alleviate symptoms, they inevitably occupy part of the nasal vestibule space, affect respiration, and worsen the sense of suffocation. Moreover, the copious watery nasal discharge associated with AR complicates the use of these products and leads to poor patient acceptance (12). Thus, developing a nasal mask that minimally affects natural breathing while providing effective allergen blocking holds significant practical value for AR patients and offers a potential novel intervention for AR prevention and treatment (9, 11, 13).

## 2 Materials and methods

### 2.1 Materials

#### 2.1.1 Fabrication of the nasal cavity model

The patients with chronic rhinosinusitis with nasal polyps (CRSwNP) included in this study met the diagnostic criteria for CRSwNP specified in *Chinese Guidelines for the Diagnosis and Treatment of Chronic Rhinosinusitis* (2018) and the diagnostic criteria for CRSwNP in *European Position Paper on Rhinosinusitis and Nasal Polyps 2020 (EPOS 2020)*, with ages ranging from 18 to 70 years. Thin-slice paranasal sinus CT scans (provided by the CT Department of Shaanxi Provincial People’s Hospital using a 128-slice spiral CT scanner, axial views from the top of the skull to the lower edge of the mandible, slice thickness = 1 mm, slice interval = 1 mm) were obtained from patients 1 month after functional endoscopic sinus surgery (FESS) for the bilateral nasal cavities. These CT images were processed using Mimics 21.0 and 3-matic 13.0 software to generate a 3D model of the “nasal cavity mold.” A 1:1 nasal sinus model, which includes the entire nasal cavity space and can be longitudinally split near the nasal septum, was fabricated using 3D printing technology.

**Figure 1:**
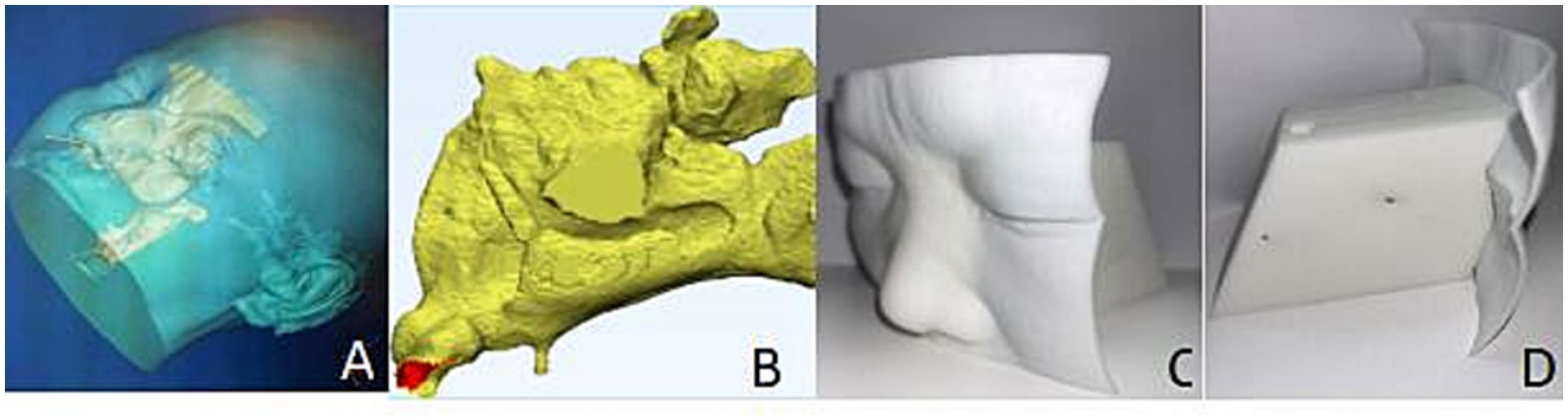
Nasal sinus model. **(A)** Cranial reconstruction; **(B)** Extraction of nasal cavity and paranasal sinus space; **(C)** 3D-printed model of the nasal cavity and paranasal sinuses; **(D)** lateral view of the model.

The computed tomography (CT) images used in this method were obtained from the Siemens SOMATOM Definition Flash dual-source CT scanner in the CT Department of Shaanxi Provincial People’s Hospital. Axial CT scans were performed on the subjects, covering the region from the top of the skull to the lower edge of the mandible, with a slice thickness of 1 mm and a slice interval of 1 mm. The experimental environment included a Windows 10 operating system, along with Mimics 21.0, 3-matic 13.0, and MATLAB R2018a software. First, the threshold analysis and boundary segmentation functions of Mimics 21.0 software were applied to rapidly construct a “mold” containing the nasal cavity space. The mold was subsequently duplicated and imported into 3-matic 13.0 software, where the smooth region marking function was used to directly “demold” and isolate the complete nasal cavity space. Finally, the isolated nasal cavity space was duplicated again and imported back into Mimics 21.0 software. The boundary segmentation function was utilized to split out the nasal cavity-sinus cavity space model, which was then duplicated and imported into 3-matic 13.0 software and saved in the STL file format for subsequent use. Through this process, accurate, complete, and rapid acquisition of the nasal cavity-sinus cavity space model was achieved (Figure 1).

#### 2.1.2 Selection of adsorbent materials

In this experiment, ultrathin dust-free paper (model: 3009A), which was cut into sizes of 0.5 cm × 0.5 cm and 4 cm × 1 cm, was selected as the APPs adsorption material.

#### 2.1.3 Preparation of dyed particles

Artemisia pollen particles (APPs) were collected from wild *Artemisia argyi* in Shaanxi Province, China. The pollen was naturally dried following multiple rounds of sampling, screening, grinding, and impurity removal. Methylene blue was chosen as the particle-staining agent. A mixture of 5 g of Artemisia extract and 1 g of methylene blue was prepared, stirred thoroughly, and then allowed to stand for 2–3 days to obtain fully stained APPs.

#### 2.1.4 Design of the pneumatic diversion barrier nasal mask

**Figure 2:**
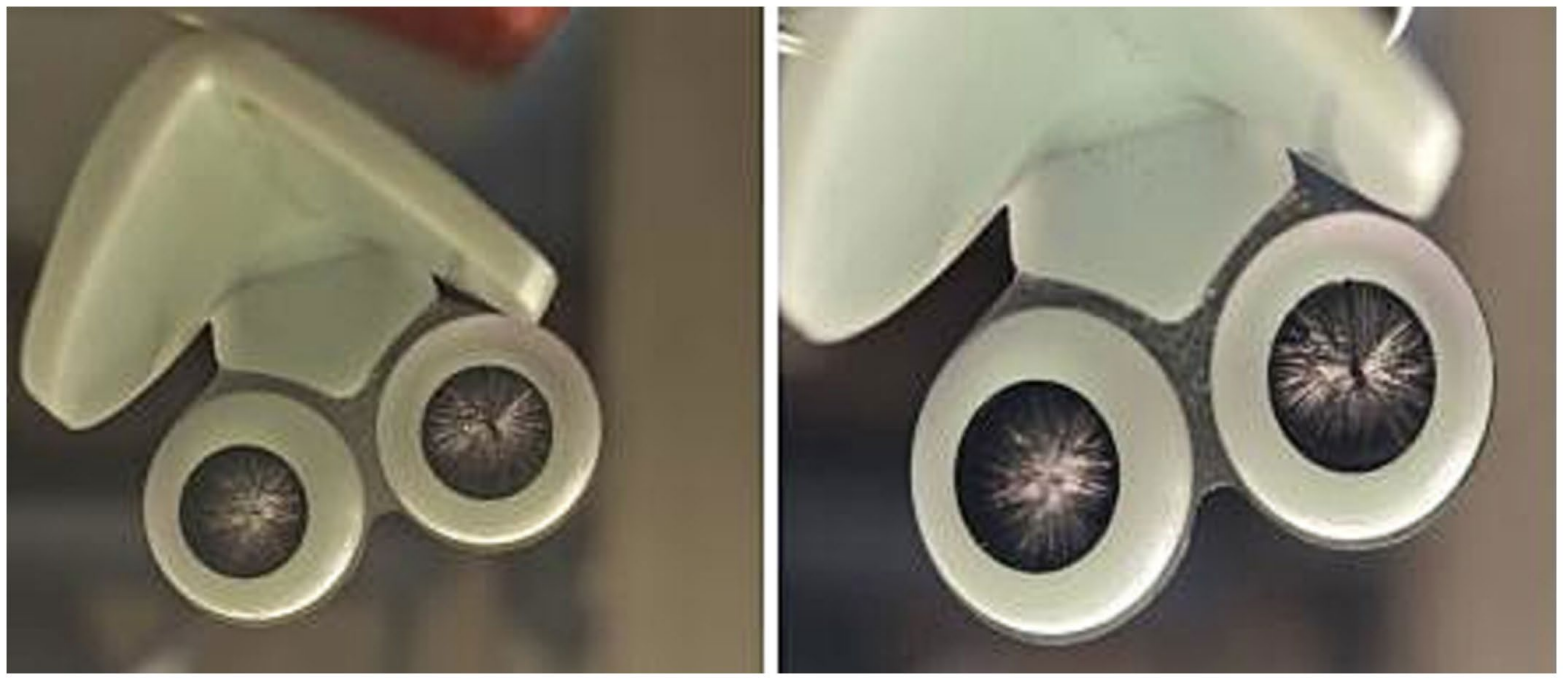
Pneumatic inflow barrier nasal mask model.

The pneumatic diversion nasal mask consists of an ergonomic triangular nasal backplate made of resin material, with a circular barrel-shaped structure (slightly larger than the anterior nostrils) attached to each side (left and right). This structure is lined with two layers of bionic nasal hairs extending from the periphery to the center and intersecting each other (Figure 2).

The bionic nasal hairs are fabricated from man-made fibers (polybutylene terephthalate) produced via melt spinning. These fibers have a diameter of approximately 0.15 mm, a curl degree of approximately 40°, and a length of approximately 1 cm, with thicker roots and tapering tips. They exhibit certain toughness and antistatic properties, essentially conforming to the characteristics of natural nasal hairs. The allergen-blocking gel (manufactured by Chengdu Bochuang Bicheng Biotechnology Co., Ltd., compliant with national standards for Class II medical devices) consists of Hypromellose and purified water, with long-chain hydrocarbons as its core component. It is safe, stable in nature, and specifically designed as an allergen-blocking gel for nasal cavity application.

### 2.2 Methods

#### 2.2.1 Experimental content

**Figure 3:**
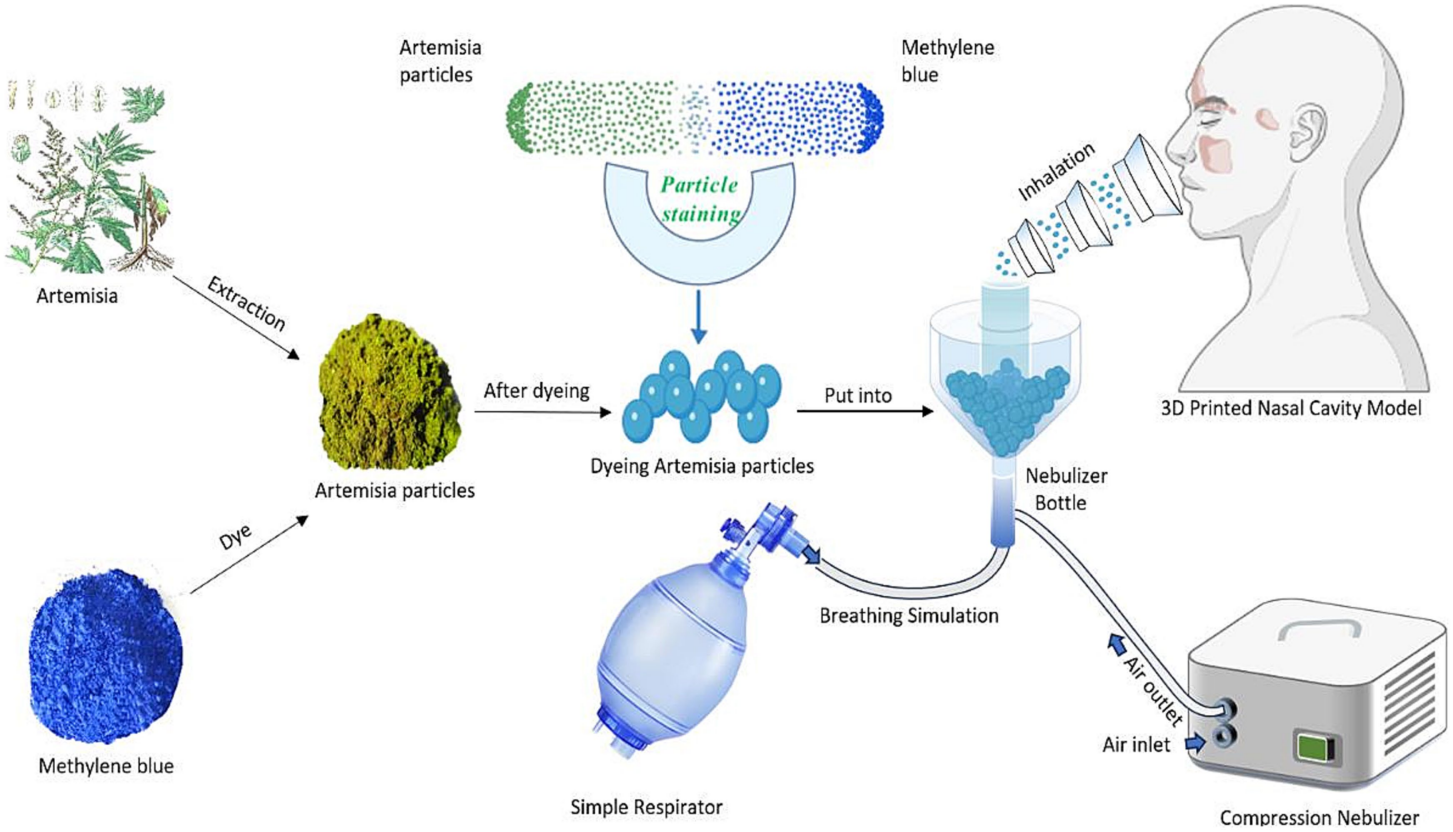
Schematic diagram of the laboratory experiment.

First, the device was connected (Figure 3). The stained Artemisia pollen particles (APPs) were placed into a nebulizer (model BM-TCC, manufactured by Hefei Qihao Medical Technology Co., Ltd.). The bottom of the nebulizer was connected to the air outlet of a compressed nebulizer via a plastic tube, the front end was connected to the nasal cavity model through a breathing mask, and the rear end was connected to a simple respirator (model FH, produced by Yangzhou Huayue Technology Development Co., Ltd.). A compressed nebulizer combined with a simple respirator was used to simulate the nebulized inhalation of stained APPs under normal conditions (16–20 breaths/min) in healthy individuals. The atomizer can ensure that the diameter of the atomized particles is greater than 5 μm, the proportion is greater than 85%, and the inhalation flow rate is 15 L/min. The barrier nasal mask could be fixed along the nasal dorsum at the front of the nasal cavity model, with the bionic nasal hair layer positioned in front of the bilateral nostrils. The allergen-blocking gel was evenly applied to the bionic nasal hair layer, with 2 sprays per layer.

Inside the nasal cavity model, 4 cm × 1 cm dust-free papers were placed on the bilateral nasal septal surfaces, and 0.5 cm × 0.5 cm dust-free papers were placed and fixed at the bilateral anterior inferior turbinate, middle inferior turbinate, posterior inferior turbinate, anterior middle turbinate, middle nasal meatus (sinus ostium), and nasopharynx.

The experiment was divided into two groups: the nonblocking group (control group) and the blocking group (experimental group). Both groups underwent simulated respiration with nebulized inhalation of stained *Artemisia annua* pollen. Each group was further divided into 15-min and 30-min subgroups, with 8 repetitions for both the control and experimental groups in each subgroup. The experiment was performed in a crossover manner between the bilateral nasal cavities. A total of 64 data points were collected.

**Figure 4:**
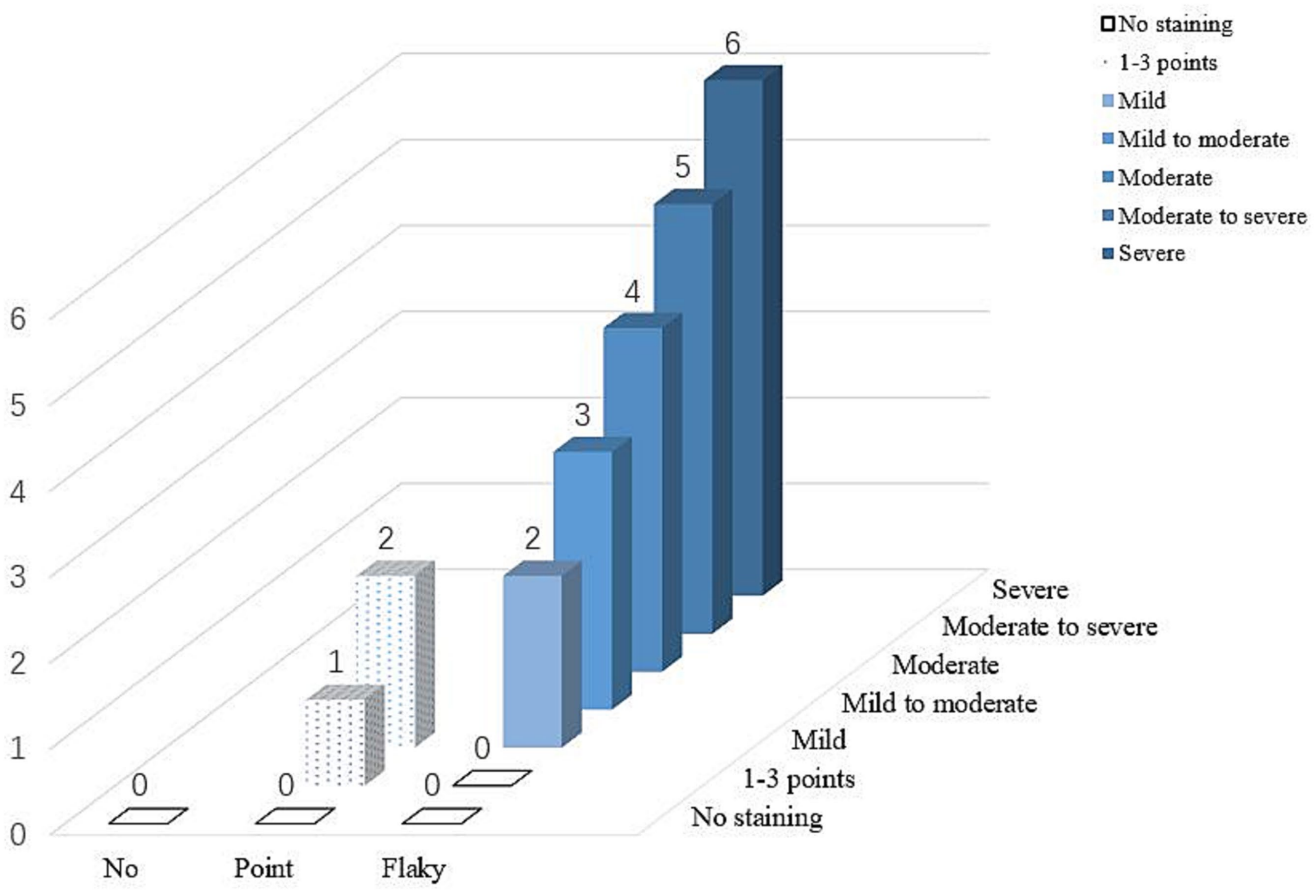
Schematic diagram of particle deposition staining score.

After each experiment, the model was quickly disassembled to observe the degree of dust-free paper staining at various sites, which was then scored. Dust-free paper with no staining was scored as 0. For a stained dust-free paper with scattered staining, 1–3 spots were scored as 1, and ≥4 spots were scored as 2. Sheet-like staining was classified into 5 grades on the basis of the degree of blue staining: mild, mild–moderate, moderate, moderate–severe, and severe, with scores ranging from 26 in sequence (Figure 4). If a dust-free paper at the same site showed different degrees of staining, the highest degree was used for scoring. A higher score indicates more APP deposition. Finally, the nasal cavity and sinus model were cleaned and dried, and the dust-free papers were replaced.

#### 2.2.2 Experimental reliability analysis

Cronbach’s alpha coefficient was used as the material method for reliability analysis in this study. The closer the Cronbach’s alpha coefficient was to 1, the more reasonable the scale design and the greater the reliability. The Cronbach’s alpha coefficient is greater than 0.8 indicates very good reliability; >0.7 means acceptable; >0.6 indicates that it should be revised but is still valuable; <0.6 requires a redesign of the item. The overall rating reliability was calculated using the following formula.


$$\alpha =\frac{K}{K-1}(1-\frac{\sum i_{2}^{S}}{x_{2}^{S}})$$

In this formula, α is the reliability coefficient, K is the number of test items, $i_{2}^{S}$ represents the score variance of all the subjects on the ith item, and $x_{2}^{S}$ is the variance of the total scores obtained by all the subjects.

#### 2.2.3 Experimental validity analysis

The validity analysis was performed using exploratory factor analysis, with the Kaiser–Meyer–Olkin (KMO) measure and Bartlett’s test of sphericity applied. The closer the KMO value is to 1, the stronger the correlation between variables, indicating that the original variables are more suitable for factor analysis: a value >0.9 indicates excellent suitability; >0.8 indicates good suitability; >0.7 indicates suitability; >0.6 indicates marginal suitability; and <0.6 indicates unsuitability for factor analysis. A *p* value <0.05 in Bartlett’s test of sphericity indicates statistical significance of the data. The calculation formula is as follows.


$$\mathrm{KMO}=\frac{\sum \sum_{i\neq j}ij_{2}^{r}}{\sum \sum_{i\neq j}ij_{2}^{r}+\sum \sum_{i\neq j}\mathrm{ij}\cdot 1,2\cdots k_{2}^{r}}$$

#### 2.2.4 Statistical methods

First, Cronbach’s alpha statistic, the Kaiser–Meyer–Olkin (KMO) value, and Bartlett’s test of sphericity were selected as indicators for reliability and validity assessment to analyze the reliability and validity of the overall scoring criteria. SPSS 28.0 software was used for data processing and analysis. Given that the data in this experiment were small-sample ordinal data, with reference to previous studies, statistical descriptions are presented as medians and interquartile ranges (P25, P75), and the Wilcoxon signed-rank test was applied for statistical analysis. A *p* value <0.05 indicated that the difference between the two groups was statistically significant.

## 3 Results

### 3.1 Results of reliability analysis and validity analysis

For the reliability analysis, Cronbach’s alpha coefficient was 0.959, which is greater than 0.9. For the validity analysis, the KMO value was 0.923, which is also greater than 0.9, and the significance coefficient of Bartlett’s test of sphericity was less than 0.001.

### 3.2 Experimental results before and after barrier construction

**Figure 5:**
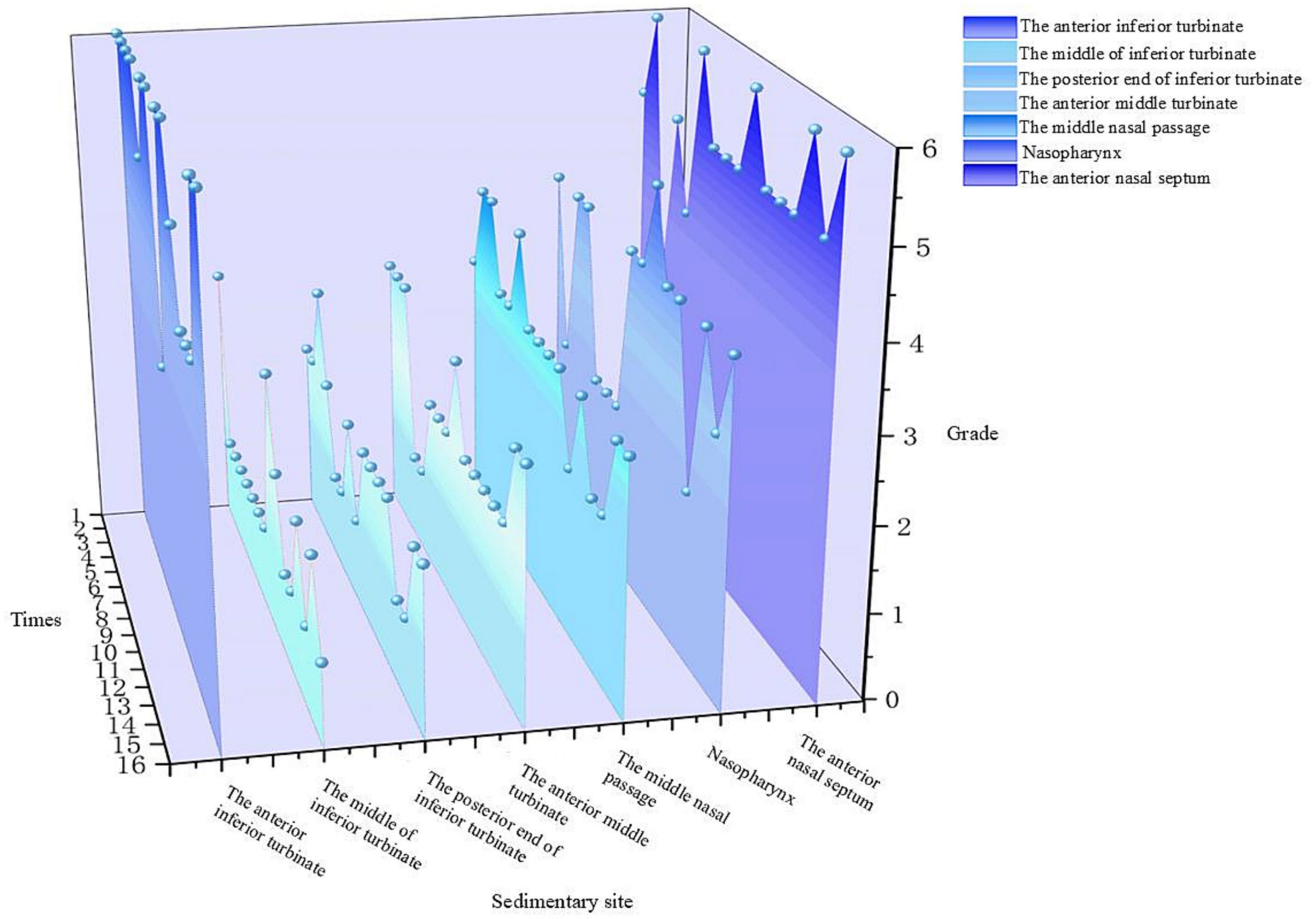
Deposition scores of various parts of the nasal cavity without a barrier nasal mask for 15 min.

**Figure 6:**
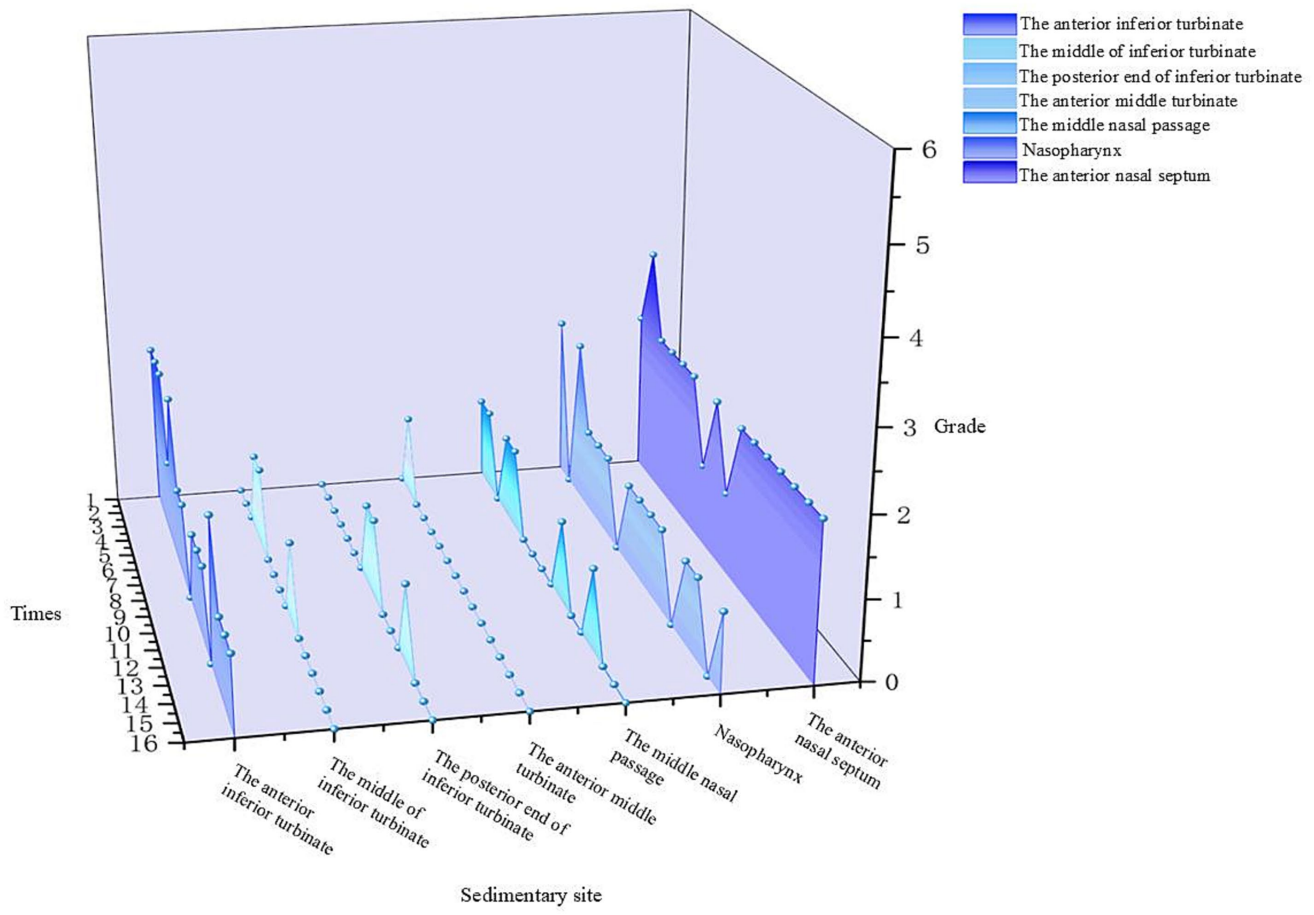
Deposition scores of various parts of the nasal cavity with a barrier nasal mask for 15 min.

**Figure 7:**
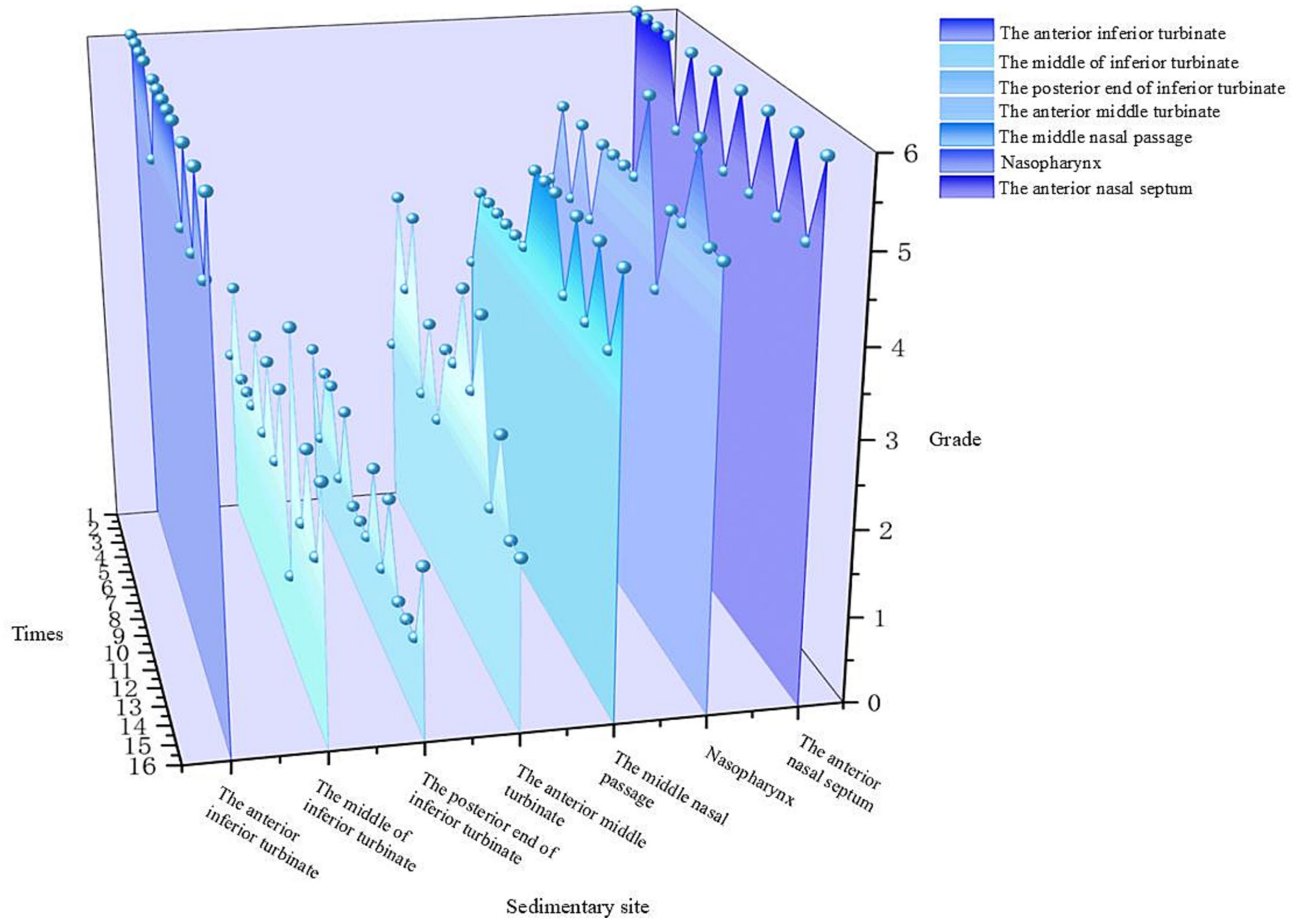
Deposition scores of various parts of the nasal cavity without a barrier nasal mask for 30 min.

**Figure 8:**
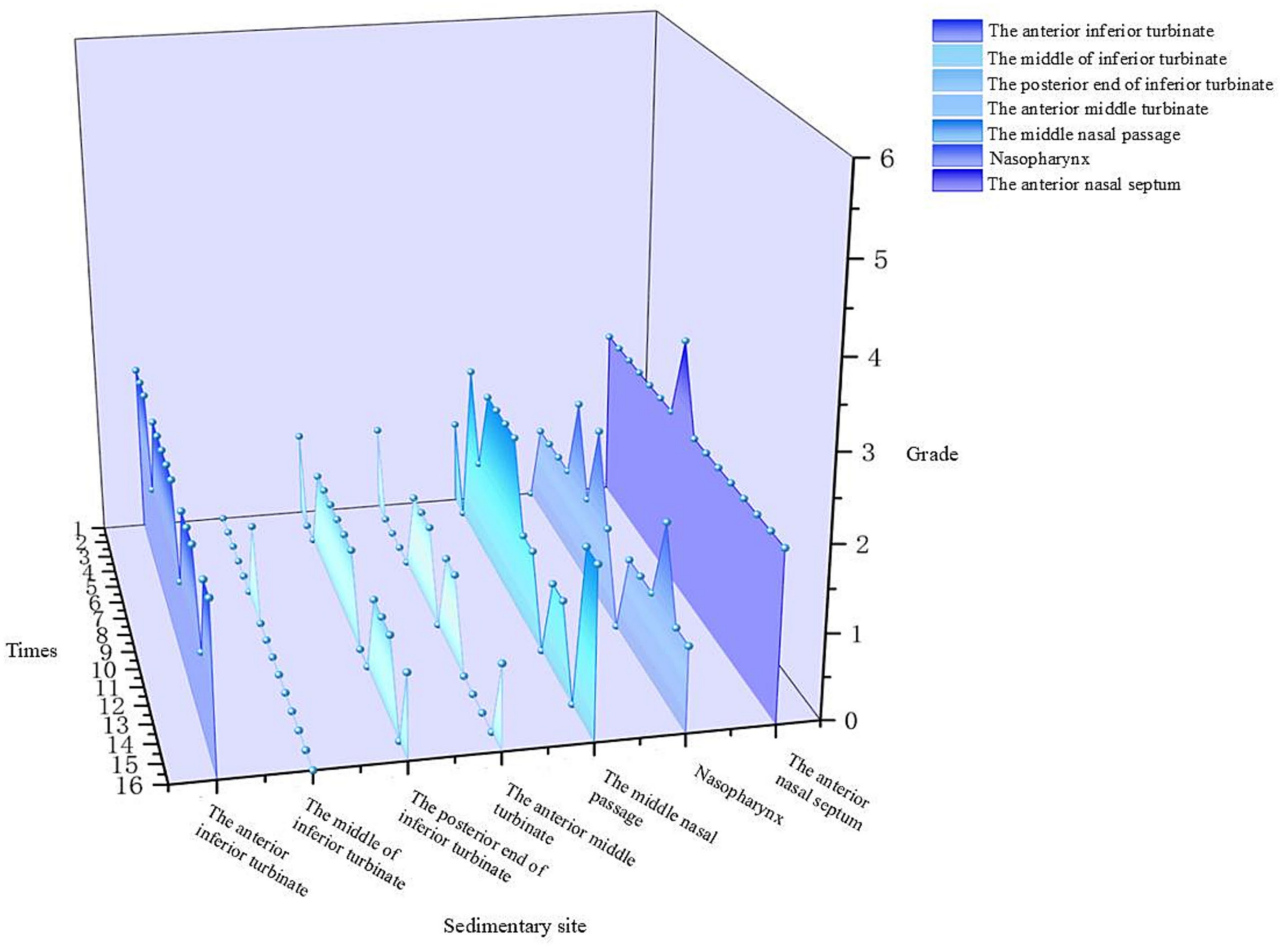
Deposition scores of various parts of the nasal cavity with a barrier nasal mask for 30 min.

After 15 min of nebulized inhalation of stained *Artemisia annua* pollen, the deposition scores of each nasal cavity site in the control group are shown in Figure 5, and those in the experimental group are shown in Figure 6. The deposition scores of each nasal cavity site in the control group after 30 min of nebulized inhalation of stained *Artemisia annua* pollen are presented in Figure 7, and those in the experimental group are presented in Figure 8. As indicated in the figures, the deposition in the control group was clearly visible at the anterior end of the inferior turbinate, the anterior part of the nasal septum, the middle nasal meatus, and the nasopharynx. In contrast, the deposition at these sites was lower in the experimental group.

The results of the Wilcoxon signed-rank test for APP deposition in various parts of the nasal cavity before and after blocking for different durations are as follows.

1. 5 min: For the total scores of all anatomical sites in the nasal cavity, the medians (interquartile range, P25, P75) of the nonblocking group and the blocking group were 3 (2, 4) and 0 (0, 1), respectively, with a Z value of −9.094 and *p* < 0.001. For the anterior end of the inferior turbinate, the medians (P25, P75) of the nonblocking group and the blocking group were 6 (4.25, 6) and 1 (1, 2), respectively, with a Z value of −3.564 and *p* < 0.001. For the middle segment of the inferior turbinate, the medians (P25, P75) of the nonblocking group and the blocking group were 1 (1, 2) and 0 (0, 0), respectively, with a *Z* value of −3.44 and *p* < 0.001. For the posterior end of the inferior turbinate, the medians (P25, P75) of the nonblocking group and the blocking group were 2 (1, 2) and 0 (0, 0), respectively, with a Z value of −3.407 and *p* < 0.001. For the anterior end of the middle turbinate, the medians (P25, P75) of the nonblocking group and the blocking group were 2 (2, 3) and 0 (0, 0), respectively, with a Z value of −3.602 and *p* < 0.001. For the middle nasal meatus, the medians (P25, P75) of the nonblocking group and the blocking group were 3 (3, 3) and 0 (0, 1), respectively, with a Z value of −3.572 and *p* < 0.001. For the nasopharynx, the medians (P25, P75) of the nonblocking group and the blocking group were 4 (2, 4) and 1 (0.25, 1), respectively, with a Z value of −3.559 and *p* < 0.001. For the anterior part of the nasal septum, the medians (P25, P75) of the nonblocking group and the blocking group were 5 (5, 6) and 2 (2, 2), respectively, with a Z value of −3.588 and *p* < 0.001 (Table 1).
2. 30 min: For the total score of the internal nasal cavity, the medians (interquartile range, P25, P75) of the nonblocking group and the blocking group were 4 (2, 5) and 1 (0, 2), respectively, with a Z value of −9.062 and *p* < 0.001. For the anterior end of the inferior turbinate, the medians (P25, P75) of the nonblocking group and the blocking group were 6 (5.25, 6) and 2 (2, 2), respectively, with a Z value of −3.598 and *p* < 0.001. For the middle segment of the inferior turbinate, the medians (P25, P75) of the nonblocking group and the blocking group were 2 (2, 3) and 0 (0, 0), respectively, with a Z value of −3.588 and *p* < 0.001. For the posterior end of the inferior turbinate, the medians (P25, P75) of the nonblocking group and the blocking group were 2 (1, 2) and 1 (0, 1), respectively, with a Z value of −3.071 and *p* < 0.05. For the anterior end of the middle turbinate, the medians (P25, P75) of the nonblocking group and the blocking group were 3 (2, 3.75) and 0 (0, 1), respectively, with a Z value of −3.548 and *p* < 0.001. For the middle nasal meatus, the medians (P25, P75) of the nonblocking group and the blocking group were 4 (4, 5) and 1 (1, 2), respectively, with a Z value of −3.555 and *p* < 0.001. For the nasopharynx, the medians (P25, P75) of the nonblocking group and the blocking group were 5 (4.25, 5) and 1 (1, 1), respectively, with a Z value of −3.63 and *p* < 0.001. For the anterior part of the nasal septum, the medians (P25, P75) of the nonblocking group and the blocking group were 6 (5, 6) and 2 (2, 2), respectively, with a *Z* value of −3.624 and *p* < 0.001 (Table 2).

**Table 1:** Comparison of nasal deposition scores of pollen particles from various anatomical sites before and after blocking for 15 min.

Anatomical sites	Group	M (P25, P75)	Wilcoxon signed-rank test	
			*Z*	*p*
Total nasal cavity	Nonblocking	3 (2, 4)	−9.094	<0.001
	Blocking-applied	0 (0, 1)		
Anterior end of the inferior turbinate	Nonblocking	6 (4.25, 6)	−3.564	<0.001
	Blocking-applied	1 (1, 2)		
Middle part of the inferior turbinate	Nonblocking	1 (1, 2)	−3.44	<0.001
	Blocking-applied	0 (0, 0)		
Posterior part of the inferior turbinate	Nonblocking	2 (1, 2)	−3.407	<0.001
	Blocking-applied	0 (0, 0)		
Anterior part of the middle turbinate	Nonblocking	2 (2, 3)	−3.602	<0.001
	Blocking-applied	0 (0, 0)		
Middle nasal meatus	Nonblocking	3 (3, 3)	−3.572	<0.001
	Blocking-applied	0 (0, 1)		
Nasopharynx	Nonblocking	4 (2, 4)	−3.559	<0.001
	Blocking-applied	1 (0.25, 1)		
Anterior part of the nasal septum	Nonblocking	5 (5, 6)	−3.588	<0.001
	Blocking-applied	2 (2, 2)		

**Table 2:** Comparison of nasal deposition scores of pollen particles from various anatomical sites before and after blocking for 30 min.

Anatomical sites	Group	M (P25, P75)	Wilcoxon signed-rank test	
			*Z*	*p*
Total nasal cavity	Nonblocking	4 (2, 5)	−9.062	<0.001
	Blocking-applied	1 (0, 2)		
Anterior part of the inferior turbinate	Nonblocking	6 (5.25, 6)	−3.598	<0.001
	Blocking-applied	2 (2, 2)		
Middle part of the inferior turbinate	Nonblocking	2 (2, 3)	−3.588	<0.001
	Blocking-applied	0 (0, 0)		
Posterior part of the inferior turbinate	Nonblocking	2 (1, 2)	−3.017	0.002
	Blocking-applied	1 (0, 1)		
Anterior part of the middle turbinate	Nonblocking	3 (2, 3.75)	−3.548	<0.001
	Blocking-applied	0 (0, 1)		
Middle nasal meatus	Nonblocking	4 (4, 5)	−3.555	<0.001
	Blocking-applied	1 (1, 2)		
Nasopharynx	Nonblocking	5 (4.25, 5)	−3.63	<0.001
	Blocking-applied	1 (1, 1)		
Anterior part of the nasal septum	Nonblocking	6 (5, 6)	−3.624	<0.001
	Blocking-applied	2 (2, 2)		

According to the above analysis, the APP deposition scores in each anatomical part of the nasal cavity significantly differed between the use of the barrier nasal mask and the nonuse of the barrier nasal mask.

The median deposition scores of each site in the nasal cavity were used to compare the conditions before and after blocking for the two usage durations (15 min and 30 min), and the blocking efficiency was calculated. The results are shown in Tables 3, 4.


$$\begin{array}{l} \;\text{Blocking efficiency}=\\ \frac{\mathrm{Pre}-\text{blocking score}-\text{Post}-\text{blocking score}}{\mathrm{Pre}-\text{blocking score}}\times 100\% \end{array}$$

**Table 3:** Deposition score and blocking efficiency of various parts of the nasal cavity before and after blocking for 15 min.

Region	15 min		Blocking efficiency (%)
	Nonblocking	Blocking-applied	
Anterior part of the inferior turbinate	6	1	83
Middle part of the inferior turbinate	1	0	100
Posterior part of the inferior turbinate	2	0	100
Anterior part of the middle turbinate	2	0	100
Middle nasal meatus	3	0	100
Nasopharynx	4	1	75
Anterior part of the nasal septum	5	2	60
Total nasal cavity	3	0	100

**Table 4:** Deposition score and blocking efficiency of various parts of the nasal cavity before and after blocking for 30 min.

Region	30 min		Blocking efficiency (%)
	Nonblocking	Blocking-applied	
Anterior part of the inferior turbinate	6	2	67
Middle part of the inferior turbinate	2	0	100
Posterior part of the inferior turbinate	2	1	50
Anterior part of the middle turbinate	3	0	100
Middle nasal meatus	4	1	75
Nasopharynx	5	1	80
Anterior part of the nasal septum	6	2	67
Total nasal cavity	4	1	75

**Figure 9:**
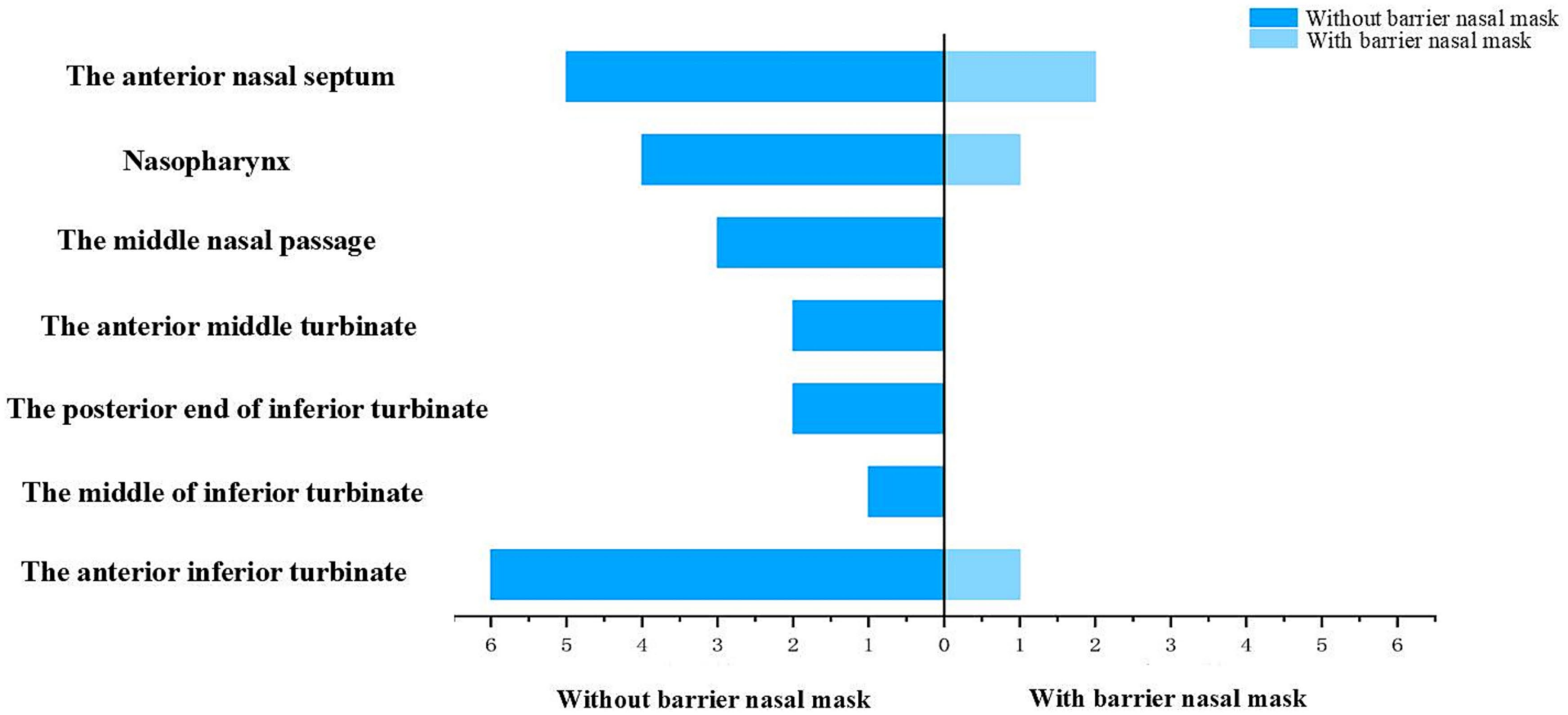
Deposition and comparison of various parts of the nasal cavity before and after blocking for 15 min.

**Figure 10:**
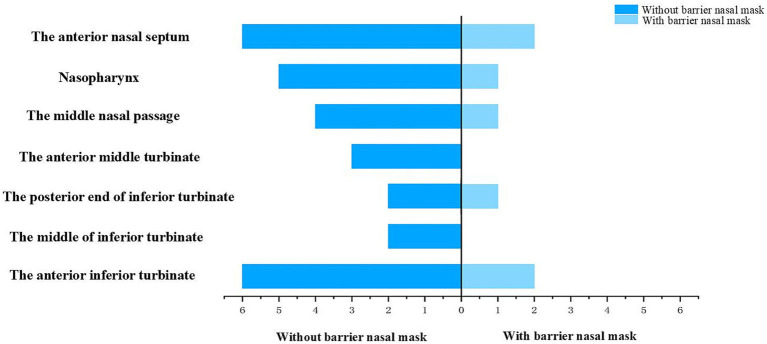
Deposition and comparison of various parts of the nasal cavity before and after blocking for 30 min.

Analysis of the median deposition scores at each site revealed that regardless of the nebulization duration (15 min or 30 min), the deposition of APP at various intranasal sites was significantly reduced when the barrier nasal mask was used. This reduction was particularly prominent at the anterior end of the inferior turbinate, middle nasal meatus, nasopharynx, and anterior part of the nasal septum (Figures 9, 10).

## 4 Discussion

This study investigated the barrier effect of a novel pneumatic inlet barrier nasal mask on pollen particles (PPs) using a 3D-printed nasal cavity model and revealed several key findings that contributed to the development of allergen protection and nasal physiology research. These findings not only address the limitations of current pollen strategies but also provide new insights into the design of personalized nasal protection devices.

### 4.1 Innovative design of the pneumatic inflow barrier nasal mask: mimicking natural nasal defense mechanisms

A key innovation of this study lies in the bioinspired integration of bionic nasal hair layers and allergen-blocking gel in the design of the nasal mask. Unlike conventional masks and nasal filters, which rely primarily on physical filtration or surface coating, the proposed nasal mask reconstructs the physiological barrier function of the nasal vestibule *in vitro*. The bionic nasal hairs, which are fabricated from antistatic polybutylene terephthalate fibers with a diameter of ~0.15 mm and a curvature of 40°, mimic the natural structure and distribution of human nasal hairs (which typically number 120–122 per nostril with lengths of 0.81–1.035 cm (14)). This design leverages the aerodynamic properties of nasal hairs, which are known to efficiently intercept particles >5 μm (15), by creating a staggered “natural filter” that disrupts laminar airflow and promotes turbulent deposition of PP. The combination of bionic nasal hairs with an allergen-blocking gel (a long-chain hydrocarbon-based gel) introduces a dual-blocking mechanism: physical interception by the hair layers and chemical adhesion by the gel. This synergy addresses the limitations of single-mechanism devices; for example, conventional N95 masks suffer from high breathing resistance (16), whereas pollen-blocking creams require frequent reapplication and may irritate the nasal mucosa (17). In our experiments, this dual mechanism achieved total blocking efficiency of 100% for Artemisia pollen particles (APP) at 15 min and 75% at 30 min (Tables 3, 4), and significantly outperformed existing nasal filters (which lack standardized filtration efficiency (18)).

### 4.2 Novel experimental model: 3D-printed postoperative nasal cavity for pollen deposition analysis

**Figure 11:**
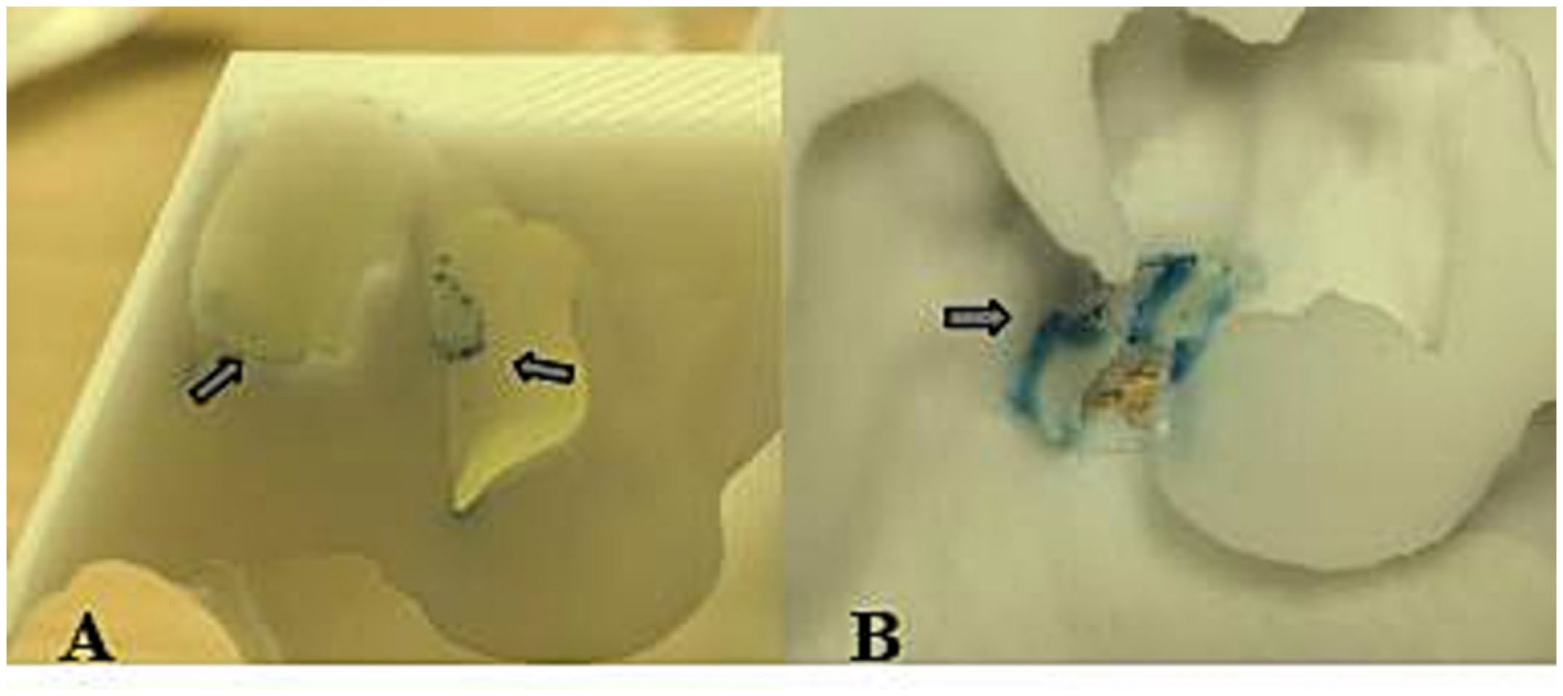
Deposition of pollen particles in the nasal sinuses at 30 min. The arrow in panel A refers to ethmoid sinus deposits, and the arrow in panel B refers to maxillary sinus deposits.

The 3D-printed nasal model was reconstructed from postoperative CT data of patients with chronic rhinosinusitis with nasal polyps (CRSwNP). This model offers several advantages over traditional *in vitro* models or numerical simulations. Unlike healthy nasal models (19), this postoperative model reflects the anatomical changes after functional endoscopic sinus surgery (FESS), such as widened sinus ostia and altered airflow patterns. Our results revealed that prolonged exposure (30 min) led to APP deposition in the maxillary and ethmoid sinuses (Figure 11), a phenomenon rarely reported in studies using healthy models. This finding highlights the importance of postoperative nasal physiology in pollen-induced exacerbations, providing a new perspective for managing CRSwNP patients with allergic rhinitis (AR). The model’s ability to be longitudinally split along the nasal septum allowed direct visualization and quantification of PP deposition in hard-to-reach regions (e.g., the nasopharynx and middle meatus) via stained adsorbent materials. This approach overcomes the limitations of numerical simulations (19, 20), which rely on computational fluid dynamics (CFD) predictions but lack experimental validation of regional deposition. Our data revealed that the highest APP deposition occurred in the anterior inferior turbinate and nasal septum, followed by the middle meatus and nasopharynx (Figures 5–8), which aligns with but extends CFD findings by confirming deposition patterns in a physical model.

### 4.3 Quantitative insights into pollen deposition dynamics: time-dependent and regional specificity

This study provides novel quantitative evidence for time-dependent and region-specific pollen deposition in the nasal cavity, which challenges existing assumptions and informs protective device design. We demonstrated that APP deposition scores increased significantly with exposure time (15 vs. 30 min) across all anatomical sites, with sinus involvement observed at 30 min. This time-dependent pattern has not been systematically documented in previous studies, which often focus on single-time-point measurements (19, 21). This finding suggests that prolonged outdoor exposure may heighten the risk of sinus inflammation in susceptible individuals, emphasizing the need for sustained protective efficacy in nasal devices. The differential blocking efficiency of the nasal mask across regions (e.g., 100% in the middle turbinate vs. 60% in the anterior septum at 15 min; Table 3) reveals that pollen deposition is not uniform and that protective devices must be optimized to target high-risk areas. This regional specificity was previously underappreciated, as most studies have evaluated overall filtration efficiency rather than site-specific protection (22, 23).

### 4.4 Bridging laboratory research and clinical applications

Pneumatic inflow barrier nasal masks represent a translational innovation by addressing unmet clinical needs in AR management. Current methods of pollen intervention have critical limitations: masks impair breathing and ocular comfort (16, 24), nasal plugs disrupt nasal physiology (18), and barrier creams require frequent application (17). Our mask, by contrast, achieves high blocking efficiency without increasing respiratory resistance (due to its biomimetic design) and avoids mucosal irritation (via external placement). The mask design, which combines bionic structures with a clinically validated allergen-blocking gel (a Class II medical device), facilitates rapid translation to clinical practice. Its demonstrated efficacy in reducing APP deposition within the middle meatus and nasopharynx—anatomical sites critical to the pathogenesis of sinusitis and asthma exacerbation (20, 25)—implies potential for mitigating not only nasal symptomatology but also lower airway comorbidities, thereby aligning with the “unified airway” hypothesis (25).

### 4.5 Limitations of the study

First, the sample size of this experiment is relatively small, and few types of PPs exist. In the future, to verify the barrier efficiency of the pneumatic inflow barrier nasal mask for pollen particles, it is necessary to increase the type of PP, use different nasal models, and obtain larger sample sizes. Second, although the 3D-printed nasal model can intuitively reproduce the internal structure of the nasal cavity, it lacks the ability to regulate mucosal blood flow and fails to simulate the effects of temperature (32–34 °C) and humidity (80–90%) on particle deposition. Moreover, the concentration of APP in the actual environment is usually lower than that designed in this experiment, so the actual barrier efficiency of the pneumatic inflow barrier nasal mask may be better than the experimental results. Third, it is necessary to select adsorption materials with higher saturation for longer-term barrier simulation experiments. Fourth, the design of the pneumatic inflow barrier nasal mask is still insufficient and needs to be improved. For example, replacing the material of the nasal back plate to increase the comfort of use; verifying the most suitable number of bionic nasal hair layers for barriers without increasing breathing resistance; and making the pneumatic inflow barrier nasal mask reusable without affecting the barrier efficiency.

## 5 Conclusion

The nasal mask used in this study is a bionic nasal mask design with a dual blocking mechanism, a postoperative 3D nasal model for realistic deposition analysis, a quantitative understanding of time- and region-dependent pollen dynamics, and a clinically translatable solution for AR patients. These findings not only deepen our understanding of pollen-nasal interactions but also provide a blueprint for the next generation of allergen protection devices. Future research will focus on optimizing the durability of masks and expanding their use to other airborne allergens to further consolidate their clinical and public health value.

## Data Availability

The original contributions presented in the study are included in the article/supplementary material, further inquiries can be directed to the corresponding author.
